# Feature identification in time series data sets

**DOI:** 10.1016/j.heliyon.2019.e01708

**Published:** 2019-05-23

**Authors:** Justin Shaw, Marek Stastna, Aaron Coutino, Ryan K. Walter, Eduard Reinhardt

**Affiliations:** a Department of Applied Mathematics, University of Waterloo, Waterloo, ON, Canada; b Physics Department, California Polytechnic State University, San Luis Obispo, CA, USA; c School of Geography & Earth Sciences, McMaster University, Hamilton, ON, Canada

**Keywords:** Time series analysis, Event detection, Feature identification, Geophysics, Oceanography, Atmospheric science, Environmental science, Geology, Hydrology

## Abstract

We present a computationally inexpensive, flexible feature identification method which uses a comparison of time series to identify a rank-ordered set of features in geophysically-sourced data sets. Many physical phenomena perturb multiple physical variables nearly simultaneously, and so features are identified as time periods in which there are local maxima of absolute deviation in all time series. Unlike other available methods, this method allows the analyst to tune the method using their knowledge of the physical context. The method is applied to a data set from a moored array of instruments deployed in the coastal environment of Monterey Bay, California, and a data set from sensors placed within the submerged Yax Chen Cave System in Tulum, Quintana Roo, Mexico. These example data sets demonstrate that the method allows for the automated identification of features which are worthy of further study.

## 1 Introduction

Geophysical researchers often study physical phenomena using instrument arrays sampling the physical variables affected by those phenomena at multiple spatial locations. This produces a data set consisting of vector time series. Features in the data set are often identified by methods such as the visual inspection of plots, or other ad hoc means. As the size and quality of geophysically-sourced time series data sets increase these methods become labor-intensive. Automated methods of identifying a set of features worthy of further study are needed.

There are an enormous variety of vector time series analysis techniques available. Empirical Orthogonal Functions (EOF) (Hannachi et al., 2007); more general dimension-reduction type methods (Pena and Poncela, 2006); wavelet (Walden and Serroukh, 2002), Fourier, harmonic, and spectral analysis methods (Emery and Thomson, 1998); data smashing (Chattopadhyay and Lipson, 2014); similarity measure approaches (Yang and Shahabi, 2004); data mining techniques (Kurbalija et al., 2010); and many more methods of varying mathematical sophistication. However, generally, existing vector time series analysis techniques are developed from a series of mathematical assumptions and then applied to data sets in a purely mathematical sense, free of physical information except for that encoded as parameters for the method. This abstraction is done both to satisfy the demands of mathematical rigor and to make the method applicable in a wide array of contexts. However, such methods apply in almost every context precisely because they largely ignore changes due to context. In particular it can become very difficult to combine the analyst's knowledge of the physical context with the interpretation of the method's output.

Many methods depend on mathematical information which may be difficult to derive from the known physical context. So for example, some methods require a choice of statistical model in order to draw comparisons (Judd et al., 2008). The results of the method depend on the statistical model chosen, but in many geophysical contexts it is not at all clear which model should be used. Moreover many statistical methods only apply to data assumed to be of a certain mathematical form, such as ergodic, steady state, etc. In many geophysical contexts it is not reasonable to adopt such assumptions on the form of the data (see for example (Mourad and Bertran-Krajewski, 2002)). Nonparametric approaches such as (Matteson and James, 2014) avoid mathematical assumptions on the form of the underlying distribution, but still use mathematical tools like cost functions whose effect on the physical interpretation of the method's output can be difficult to determine. Even if certain mathematical assumptions are appropriate in a given context, not all researchers will have the background necessary to encode their knowledge of the physical context in a statistical model. If the researcher does not know what part of the method's output is from the physics, and what part is from the underlying mathematics, their confidence in deriving conclusions about the physics will be severely limited. Finally, for practical purposes, more advanced data analysis methods are often limited in their usefulness by the availability of user-friendly software (e.g., the open and widely used package by (Torrence and Compo, 1998)). The method we present ameliorates all the concerns just listed, because it uses the researcher's knowledge of the physical context without requiring them to quantify it for use in a mathematical formalism.

One may rebut the concerns just outlined by pointing out that standard methods in geosciences could be used because their physical interpretations have been made clear over time through widespread use. However familiar methods are not well suited to identifying features in vector time series caused by physical phenomena. For example EOF-type methods (Hannachi et al. (2007)) can process such data sets, but the focus here is on identification of events whose time duration is much shorter than the total record. EOFs are variance-maximizing, and while high total variance in a mode may be the result of an event, it may also be the result of low variance over the entire record. Methods of this type are therefore ill-suited for event detection. Similarly methods for comparing two time series abound, e.g. correlation, covariance, or coherence (Emery and Thomson, 1998); (Torrence and Compo, 1998), but when these methods are applied pairwise to a data set with more than two series there is a combinatorial explosion of options: if there are *k* series, there are $\left(\begin{array}{c} k\\ 2 \end{array}\right)=k(k-1)/2$ such pairs. There are algorithms that address this issue (Lyubushin, 2018a) but the sophistication of the mathematics ramps up quickly. The method presented here can be applied to any number of time series simultaneously, subject only to memory constraints.

The purpose of this paper is not to downplay the value of existing methods, but rather to present a method for those researchers who would gladly trade some mathematical sophistication for a clearer link with the known physical context and a lower implementation cost. We present a physics-based, computationally inexpensive, flexible, easily-implemented, and transparent method for the automated identification of features caused by physical phenomena. We call this method ‘the *γ* method,’ and it is outlined in Section 2. In section 3 the method is applied to a data set from the coastal environment of Monterey Bay, California (section 3.1), and a data set from the Yax Chen Cave System, near Tulum, Mexico (section 3.2). Section 4 includes further discussion. The supplementary material includes tutorial codes for the *γ* method written in MATLAB, R, and python.

## 2 Methods

### 2.1 The *γ* method

Before details are presented we outline the *γ* method in broad terms. To streamline the presentation we assume that the data has been controlled for quality and filtered by whatever methods the discipline deems appropriate. Assume the data set consists of time series $\left\{x_{1}(t),x_{2}(t),\ldots ,x_{k}(t)\right\}$ sampling multiple physical quantities with sensors nearby one another, as they would be in a single instrument cluster. We expect that physical phenomena of interest will impact multiple physical quantities nearly simultaneously. For example, Fig. 4A of (Maio et al., 2016) shows tropical storm Irene affecting wind speeds and air pressure as it passes a meteorological station. The physical quantities impacted by an event lead to deviations from the background state in the associated time series (wind speed and pressure in this case). We have now formulated the problem:

Problem Statement 1 *Given a data set consisting of time series* $\left\{x_{1}(t),x_{2}(t),\ldots ,x_{k}(t)\right\}$*, identify time periods (features) denoted* $\left\{F_{1},F_{2},\ldots \right\}$ *in which all* $x_{i}(t)$ *experience a deviation from their respective trends.*

To solve this problem, we proceed as follows. For each time series $x_{i}(t)$, form the associated absolute deviation series

(1) $${\overset{ˆ}{x}}_{i}(t)=\kappa_{i}|x_{i}(t)-\mu_{i}(t)|$$

where $\kappa_{i}$ is a scaling constant and $\mu_{i}(t)$ is some trend chosen by the analyst as appropriate to the physical context. Large values of ${\overset{ˆ}{x}}_{i}$ correspond to large deviations from the trend, and small values correspond to values of $x_{i}$ near the trend. Absolute deviation rather than standard deviation is used to avoid accentuating outliers. The absolute deviation series is still affected by outliers, but accentuates them less than the corresponding standard deviation series. For an in-depth discussion see (Huber and Ronchetti, 2009). Features in the data set are identified using the maxima of the time series

(2) $$\gamma (t)=\min_{i}\{{\overset{ˆ}{x}}_{i}(t)\}=\min_{i}\{\kappa_{i}|x_{i}(t)-\mu_{i}(t)|\}$$

at every time *t* (note that γ(t)≥0). We will call the set of time series $\left\{x_{i}\right\}$ included in the definition of γ(t) the ‘defining set’ of time series for γ(t). Notice also that by construction of *γ*, any number of time series may be in the defining set, so this method is not a pairwise comparison method.

The key observation is this: because γ(t) is defined as the minimum curve, if it is perturbed from zero, all curves are perturbed from zero. Therefore, if we wish to find times where all time series are experiencing deviations from their respective trends, we should look for deviations in γ(t). In particular, the maxima of γ(t) correspond to times when all physical quantities sampled by the time series in the defining set are experiencing large deviations from their respective trends. Following the reasoning above we expect these deviations to be caused by some physical phenomenon. Although each physical variable will not be perturbed at exactly the same time or for the same duration, we expect some time overlap of deviations in affected fields. The *γ* method identifies such times (see the Figures in section 3). Time periods near these maxima are defined as features of interest for further study. Arranging the maxima in descending order produces a rank-ordered set of time extents as identified features $\left\{F_{1},F_{2},\ldots \right\}$, where the ranking is essentially by size of overlap. See the accompanying tutorial codes for a constructed example.

By construction this set of features is dependent on the choice of defining set, which allows tuning of the method for specific phenomena. The analyst uses their knowledge of the physical context to decide which time series to include in the defining set, an appropriate trend, and how to synchronize the time series to one another. The chosen time series must then be scaled so that they may be compared in γ(t). Finally, the feature length must be chosen. We consider each step in turn.

### 2.2 The defining set

The defining set can be chosen any way the analyst sees fit. If the analyst is looking for a specific physical phenomena, only the fields whose deviations would be associated with those events are included in the defining set. Alternatively the method may be applied to various subsets of the available time series to identify features first, with the analyst supplying physical explanations afterward.

The analyst may construct any time series they deem useful and include it in the defining set. For example, suppose two thermistor chains are deployed in a small lake. The thermistor chains each produce a vertical vector of temperature time series. If all temperature time series are included in the defining set the corresponding γ(t) has maxima when there is a temperature deviation at all sensors simultaneously. This choice of defining set may identify periods of temperature change driven at the lake scale, such as a deviation of temperature due to seasonal change. If instead the phenomena of interest ia a cold water inflow, it may suffice to take the depth-averaged values at each chain and consider the difference of the two averaged time series as an indicator. Any time series the analyst can think of, and whose deviation would serve as an indicator for the given physical context and problem, may be included in the defining set. This would include smoothed versions of existing time series which preserve the relevant deviations (Rong and Bailis, 2017), as well as time series produced from standard methods like EOF (i.e. amplitude time series) and scale-averaged wavelets if the analyst deems it appropriate (Walter et al., 2017).

Once the defining set is chosen, a trend must be chosen for each time series. If the trend is unknown, mathematical methods such as (Wu et al., 2007) may be used to identify it, but this is not always necessary. The time mean $\mu_{i}(t)={\langle x_{i}(t)\rangle }_{t}$ is a reasonable constant valued choice in many applications. This is the choice we make for both data sets in section 3.

Finally, the defining set must be synchronized. Different sensors may have different sampling rates, deployment duration, etc. The analyst uses their knowledge of the instruments and physical context to arrange the time series from each sensor along some global time regime. This global time regime *t* is the time on which γ=γ(t) depends. Differences in sampling rate may be handled by interpolation or subsampling, differences in duration by truncation to an appropriate overlapping time period, and so on. Once the defining set has been chosen and synchronized, the scaling must be chosen.

### 2.3 Scaling

Equation (1) includes a scaling constant for each absolute deviation series for two reasons. First, equation (2) defines γ(t) as the minimum of all absolute deviation time series at every point in time. For this to make any physical sense every time series in the defining set should be nondimensionalized because each of them are sampled from physical quantities having possibly different units. Second, the choice of nondimensionalization constant $\kappa_{i}$ allows further tuning of the method. Scalings may be chosen to increase the influence of some physical quantities on γ(t) while decreasing the influence of others. For the examples given in section 3 we have chosen to scale each time series by their respective maximum values. In general, the choice of scaling is another opportunity for the analyst to apply their knowledge of the context and tune the *γ* method to their purposes.

### 2.4 Feature length

Once the analyst has chosen the defining set, trend, synchronization, and scalings, the final choice is feature length *l*. This parameter is simply an approximate length of time that the physical phenomena of interest is expected to last. In our algorithm, we use a windowing procedure, where maxima of *γ* are identified, and features are defined as the time window of length *l* whose midpoint is at the maxima. If the feature length is unknown, then *l* may be set to be very short so that features identify maxima in *γ*.

### 2.5 Feature identification

The work in previous sections allows us to write Problem 1 as:

Problem Statement 2 *Given a defining set consisting of time series* ${{\left\{x_{i}(t)\right\}}_{i=1}}^{k}$ *synchronized along a global time regime, with respective scaling constants* $\kappa_{i}$ *and trends* $\mu_{i}(t)$*, form*

$$\gamma (t)=\min_{i}\{\kappa_{i}|x_{i}(t)-\mu_{i}(t)|\}.$$

*Identify rank-ordered features* $\left\{F_{1},F_{2},\ldots ,F_{r}\right\}$ *as time windows of length l centered at the local maxima of γ.*

We solve this problem iteratively, allowing overlapping features. Note that this means, for example, that the top several maxima of *γ* may all be included in the first feature. In that case the second feature would not be centered at the second highest global maximum, but rather at the highest maximum outside the first feature.

Problem 2 is solved using Algorithm 1. The rank-ordered identified features $\left\{F_{1},F_{2},\ldots \right\}$ are generated by iteration on the maxima of γ(t). MATLAB codes implementing Algorithm 1 were used for all results presented in section 3. Tutorial codes in MATLAB, R, and python are included in the supplementary material.

**Algorithm 1:**
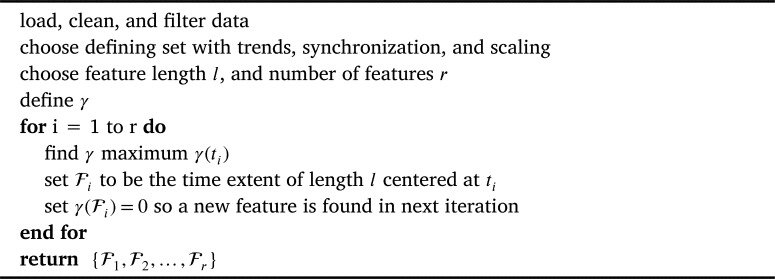
Identify Features

## 3 Results

### 3.1 Monterey Bay

The first data set we will consider is from a moored array of instruments deployed in the nearshore coastal environment of Monterey Bay, California from July 7–21, 2011. The moored array measured density (derived from temperature and conductivity measurements) and velocities throughout the water column. For a detailed analysis of this data set see Walter et al. (2016). High-resolution measurements were collected near a persistent upwelling front that forms between recently upwelled waters and warmer stratified waters that are trapped inside the bay (termed an upwelling shadow front). The front propagates as a buoyant plume front past the instrument array with high kinetic energy before breaking up into a combination of large amplitude internal waves and instabilities.

Both density *ρ* and kinetic energy $KE=\frac{1}{2}\left(u^{2}+v^{2}+w^{2}\right)$ (omitting $\rho_{0}$) are useful for identifying fronts, internal waves, and instabilities. The overlap of the time series of both quantities has dimensions M×N=35×19701 where *M* is the number of points in depth *z*, binned 0.5 m apart, and *N* is the number of samples in time *t*, taken every minute. Each of the vector-valued time series for *ρ* and *KE* are comprised of 35 time series, for a total of 70 individual time series. The *γ* method may be applied directly to these 70 series, but a much simpler choice is appropriate in this context. The large kinetic energy and density events of interest tend to induce changes in the whole portion of the water column sampled by the data set. This makes the depth averaged means $\bar{\rho }$ and $\bar{KE}$ good indicators. These are 2 time series of length *N*, and we take them as our defining set. These time series are already synchronized because we expect fronts, internal waves, and instabilities to cause deviations in *ρ* and *KE* nearly simultaneously. We also scale each of the deviation series by their maximum values since we consider both to be equally important. These choices then define γ(t). Based on known forcing associated with local diurnal winds (cf. (Walter et al., 2016)), we define our feature length as a day.

**Figure 1:**
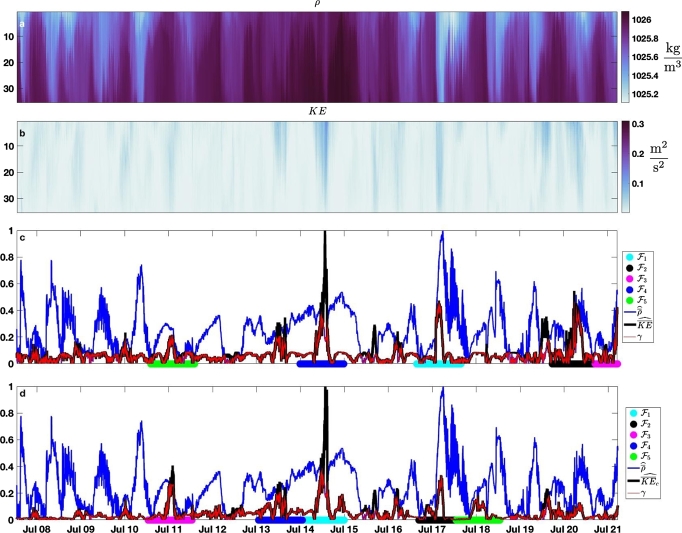
The *γ* method applied to the Monterey Bay data set. Panel a shows the full density *ρ* (kg/m^3^) and panel b shows the full kinetic energy *KE* (m^2^/s^2^). In both a and b the vertical axis is bin number. Panel c shows the results of the *γ* method using the defining set $\left\{\bar{\rho },\bar{KE}\right\}$, and panel d shows the results of using the *γ* method using the defining set $\left\{\bar{\rho },{\bar{KE}}_{c}\right\}$. All panels are aligned along the global time regime indicated below panel d.

Fig. 1 panel c shows the result of applying the *γ* method. Panel c shows the first five features $F_{i}$. Notice the most important feature, $F_{1}$, corresponds to the frontal crossing of July 17, a feature identified and studied extensively in (Walter et al., 2016). In (Walter et al., 2016), this particular event was identified based on a more complicated filtering and wavelet analysis of the data set. Features $F_{2}$ and $F_{3}$ are large frontal crossing and internal wave events, and $F_{4}$ coincides with a large regional-scale upwelling event and delineates a difference in forcing relative to earlier events (see discussion in (Walter et al., 2016)). The next most important feature is $F_{5}$. The density profile, along with the velocity data (not shown) indicates that this feature is an across shore pulse of cold water (see (Walter et al., 2016) Fig. 1 b for orientation of axes). This is an example of a feature which may not have been identified by an analysis that did not use the method.

Fig. 1 panel d shows the result of applying the *γ* method using $\bar{\rho }$, and an alternate choice of a second time series. Stratification stabilizes the water column. When kinetic energy is high but stratification is weak, we expect more vertical mixing. To capture this idea, we define the conditioned depth averaged kinetic energy, ${\bar{KE}}_{c}$ as

(3) $${\bar{KE}}_{c}=\frac{\bar{KE}}{\left|\rho_{B}-\rho_{T}\right|}$$

where $\rho_{B}$ is the density at the bottom sensor, and $\rho_{T}$ is the density at the top sensor. ${\bar{KE}}_{c}$ is larger when the stratification is weak. The defining set is $\left\{\bar{\rho },{\bar{KE}}_{c}\right\}$. Applying normalization by the maximum as before defines γ(t), leading to the results shown in Fig. 1 panel d. Note that $F_{1}$ is now the upwelling period from July 14th to 15th. The large frontal crossing on July 17 is still identified as $F_{2}$. This shows that important features may persist under time series conditioning. The across shore pulse of cold water is now identified as $F_{3}$, because stratification is weak during this period. $F_{4}$ is also a newly identified feature that is likely driven by strong surface wind forcing, due its confinement to the near-surface region. Finally, $F_{5}$ identifies a time when $\bar{KE}$ is small, but the stratification is weak and the water is cold: this is another weakly stratified cold water pulse. Both cold water events $F_{3}$ and $F_{5}$ are not immediately clear from panels a or b of Fig. 1, because the eye is drawn to the other events (see (Wang et al., 2004) for a discussion of the human visual system). In this way the *γ* method identifies features previously identified by analysts, but may also identify features that analysts miss.

### 3.2 Yax Chen

For the second example, we apply the *γ* method to a data set from the submerged Yax Chen Cave System, in Tulum, Quintana Roo, Mexico. The Yax Chen Cave System is part of the larger Ox Bel Ha Cave System. The data set consists of time series from pressure (*p*), conductivity (*s*), and temperature (*T*) sensors deployed within Yax Chen from May 2016 to April 2018. The sensors were deployed as a follow up to the work presented in Coutino et al. (2017) in order to observe the changes in the aquifer as a result of heavy rainfall events from hurricanes and tropical storms, which are common to the region. The sensors were deployed 10 m downstream from a cenote at a depth of 4 m. There was a single sensor for each physical quantity, and the three sensors sampled simultaneously every 30 minutes, so the time series are synchronized. Each time series has dimensions M×N=1×33697 so there is no need to reduce the spatial dimension in this case. Normalization is taken by the respective maxima, and the feature length as one week.

**Figure 2:**
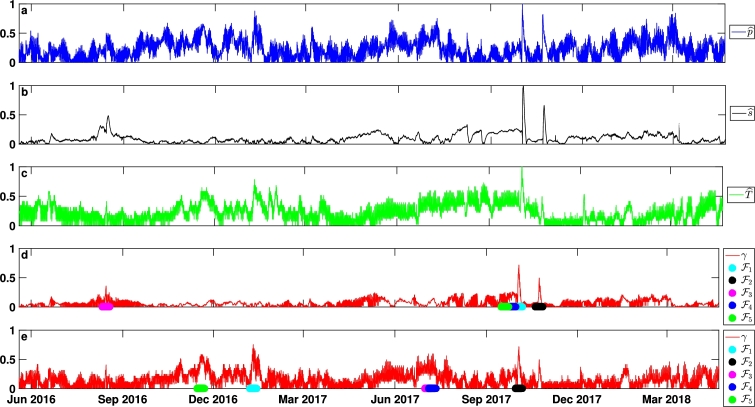
The *γ* method applied to the Yax Chen data set. Panel a shows $\overset{ˆ}{p}$, panel b shows $\overset{ˆ}{s}$, and panel c shows $\overset{ˆ}{T}$. Panel d is *γ*(*t*) for the defining set {*p*,*s*,*T*}. Panel e is *γ*(*t*) for the defining set {*p*,*T*}.

Fig. 2 panel d summarizes the results of applying the *γ* method using the defining set of {p,s,T}. The early October 2017 event, corresponding to hurricane Nate^1^ is identified as $F_{1}$. The late October event, corresponding to hurricane Philippe is identified as $F_{2}$. The mid August event corresponds to hurricane Earl, identified as $F_{3}$. The last two features $F_{4}$ and $F_{5}$ identify the time period from mid to late September in which several storms, including hurricanes Irma and Jose could still have been affecting changes in the parameters measured in Yax Chen. This choice of the defining set identifies rainfall events large enough to affect pressure, salinity, and temperature in the cenote.

Fig. 2 panel e summarizes the results of applying the *γ* method using the defining set of {p,T}, i.e. without salinity. Since variations in salinity can only be due to mixing with the underlying marine water, this choice of defining set allows for the identification of events associated with longer trends, as opposed to turbulent mixing events (Coutino et al., 2017). Features $F_{1}$ (early January 2017) and $F_{5}$ (mid November 2016) correspond to large rain events that are not hurricane related. Early October 2017, $F_{2}$, corresponds to hurricane Nate. A hurricane's primary expression in the cave network is via the turbulent mixing between the meteoric lens and the underlying marine water mass, resulting in variations in *s*, but *s* is not included in the defining set. This explains why hurricane Nate is not identified as $F_{1}$, and also why Hurricane Phillippe is not captured. Features $F_{3}$ and $F_{4}$ (first half of July 2017) do not coincide with large rainfall events, and their identification by the *γ* method as epochs which merit further study is completely new.

## 4 Discussion & conclusions

Section 3.1 shows that the *γ* method is able to automatically identify features of interest previously identified in an ad hoc manner, while also identifying new significant events. This means the *γ* method can be applied to previously studied data sets and may find new results. Section 3.2 shows that the *γ* method may be applied as soon as the physical context is known, to identify a set of features worthy of further study. Both examples outline how the analyst uses their knowledge of the physical context to choose the defining set, trend, scalings, synchronization, and feature length. For the sake of presentation we have outlined a broad range of possible necessary steps for choosing and synchronizing the defining set. However, the practical application of the *γ* method to a particular data set needs only a few steps. In practice we have found that taking the trend set to be the time mean and scaling by respective maxima serve as good default choices.

The *γ* method depends on the overlap of perturbed fields. For short-duration features, or time series from sensors spaced far apart, it may be beneficial to time lag the time series before applying this method. For example, using the example of two thermistor chains in a lake from section 2.2, if the analyst is interested in temperature changes due to inflow, water masses inducing the change in temperature may pass the two thermistor chains separated by some time lag. In this case it may be preferable to make the defining set to be all of the sensors, but with an appropriate time lag on time series from one of the chains. If time lags are unknown but suspected, it may be possible to infer them by brute force application of the *γ* method to a range of possible time lags. Finding the time lag appropriate for a given time series is a highly field- and application-dependent problem and so must be left to the analyst, or other methods.

If the knowledge of the physical context is incomplete, so that expected phenomena or time lags for synchronization are unknown, a modified version of the method may still be applied as follows. The defining set should include many, if not all, of the available time series. Since the phenomena and time lags are unknown, it may be that a feature of interest perturbs some but not all time series at a given time. The *γ* method presented above is inappropriate, because a single time series being unperturbed will cause the method to miss the feature altogether. There is a simple fix for this: define γ(t) not as the pointwise minimum of the deviation series (equation (2)), but as some suitable intermediary curve. For example if the method is applied to a defining set with 10 time series, it is probably worth investigating features which result from the deviation of 8 of them, so γ(t) could be taken as the third from minimum curve. Taking an intermediary curve for γ(t) also ameliorates the problem of faulty or intermittent sensors. Note this modification essentially ignores time series whose time lags cause them to be unsynchronized with the rest of the data set. The level of the intermediary curve is another parameter that may be swept. In general, the weaker the knowledge of the analyst, the more parameters there are to sweep. The code runs on the order of seconds on modest hardware on all data sets we have tried, and is easy to parallelize for larger data sets or large sweeps, as necessary.

There are many other immediate possible extensions of the *γ* method. If positive and negative deviations from the mean are not equally important, the definition of *γ* may be changed to a signed deviation instead. If the data is streaming rather than complete, the method could be applied with a trend *μ* defined by an appropriate recent window, resulting in an analogue of more sophisticated methods such as those presented in (Hill and Minsker, 2010). Features could be chosen by looking for extended deviations of *γ*, rather than maxima. The most likely next application of the method for our research will be to apply it to time series pulled from numerical experiments in order to identify temporally under-resolved subsections which need to be rerun. The reader may have noticed any number of immediate modifications that could be made to the method as it was presented.

Hurricane Nate's identification over both choices of defining set in section 3.2 suggests that the *γ* method could be employed to identify important features by their persistence across choices of defining set. Persistence over a parameter sweep is used as a measure of a topological feature's importance in topological data analysis (see section 2.4 of (Chazal et al., 2015) for an intuitive explanation). The *γ* method could be run multiple times to sweep the choice of defining set as the parameter, yielding a final output of the most frequent features across all choices of the defining set. These persistent features would then be candidates for closer study.

Clearly the *γ* method is not as mathematically sophisticated as some other options. It is not designed to outline spectral information, identify weak synchronous signals, or automatically identify correct time shifts or choose the correct scaling. More sophisticated methods such as (Lyubushin, 2018b) address all of these concerns. However, even those readers with the resources to confidently apply one of the many vector time series methods available to yield results they are satisfied with may find the *γ* method useful as a diagnostic. In many cases we have found that the *γ* method's incredible clarity and speed make it worth running before more sophisticated methods. For example the *γ* method may be used to define time periods in a data set on which other methods are applied. Continuing the lake example, the method could be used to identify features defining cold and warm time periods before applying conventional methods to the data within those time periods. The results of the conventional methods may then be compared and contrasted across different time periods. The advantage of this process is that the time periods are defined mathematically, rather than by visual inspection.

In summary, the implementation of the *γ* method to a given data set is straightforward and computationally inexpensive. The method is flexible and transparent, which allows it to be employed in a wide variety of contexts, and easily modified as necessary. After the initial tuning of the choices for a given context and problem, the method automates identification of a set of features which are worthy of further study.

## Declarations

### Author contribution statement

Justin Shaw: Conceived and designed the experiments; Performed the experiments; Analyzed and interpreted the data; Wrote the paper.

Marek Stastna: Conceived and designed the experiments; Performed the experiments; Analyzed and interpreted the data; Contributed reagents, materials, analysis tools or data; Wrote the paper.

Aaron Coutino, Ryan K. Walter: Analyzed and interpreted the data; Contributed reagents, materials, analysis tools or data; Wrote the paper.

Eduard Reinhardt: Analyzed and interpreted the data; Contributed reagents, materials, analysis tools or data.

### Funding statement

This work was supported by an NSERC grant (RGPIN-311844-37157) and a PGS-D.

### Competing interest statement

The authors declare no conflict of interest.

### Additional information

Supplementary content related to this article has been published online at https://doi.org/10.1016/j.heliyon.2019.e01708.

No additional information is available for this paper.

## Supplementary material

The following Supplementary material is associated with this article:

GAMMA_TUT.R Gamma Method Tutorial Code. GAMMA_TUT.R

GAMMA_TUT.M Gamma Method Tutorial Code. GAMMA_TUT.M

GAMMA_TUT.PY Gamma Method Tutorial Code. GAMMA_TUT.PY

## Footnotes

1 All hurricane dates retrieved from the National Hurricane Center (https://www.nhc.noaa.gov/).

## References

[br0010] Chattopadhyay I., Lipson H. (2014). Data smashing: uncovering lurking order in data. J. R. Soc. Interface.

[br0020] Chazal F., Glisse M., Michel B. (2015). Convergence rates for persistence diagram estimation in topological data analysis. J. Mach. Learn. Res..

[br0030] Coutino A., Stastna M., Kovacs S., Reinhardt E. (2017). Hurricanes Ingrid and Manuel (2013) and their impact on the salinity of the Meteoric Water Mass, Quintana Roo, Mexico. J. Hydrol..

[br0040] Emery W.J., Thomson R.E. (1998). Data Analysis Methods in Physical Oceanography.

[br0050] Hannachi A., Jolliffe I., Stephenson D. (2007). Empirical orthogonal functions and related techniques in atmospheric science: a review. Int. J. Climatol..

[br0060] Hill D.J., Minsker B.S. (2010). Anomaly detection in streaming environmental sensor data: a data-driven modeling approach. Environ. Model. Softw..

[br0070] Huber P., Ronchetti E. (2009). Robust Statistics.

[br0080] Judd C.M., McClelland G.H., Ryan C.S. (2008). Data Analysis: A Model Comparison Approach.

[br0090] Kurbalija V., Radovanović M., Geler Z., Ivanović M. (2010). A Framework for Time-Series Analysis.

[br0100] Lyubushin A. (2018). Global coherence of GPS-measured high-frequency surface tremor motions. GPS Solut..

[br0110] Lyubushin A. (2018). Synchronization of Geophysical Field Fluctuations. Complexity of Seismic Time Series.

[br0120] Maio C.V., Donnelly J.P., Sullivan R., Madsen S.M., Weidman C.R., Gontz A.M., Sheremet V.A. (2016). Sediment dynamics and hydrographic conditions during storm passage, Waquoit Bay, Massachusetts. Mar. Geol..

[br0130] Matteson D.S., James N.A. (2014). A nonparametric approach for multiple change point analysis of multivariate data. J. Am. Stat. Assoc..

[br0140] Mourad M., Bertran-Krajewski J.L. (2002). A method for automatic validation of long time series of data in urban hydrology. Water Sci. Technol..

[br0150] Pena D., Poncela P. (2006). Dimension reduction in multivariate time series. Advances on Distribution Theory, Order Statistics and Inference, in Honor of B.C. Arnold. 1981.

[br0160] Rong K., Bailis P. (2017). ASAP: prioritizing attention via time series smoothing. Proceedings of the VLDB Endowment 10.

[br0170] Torrence C., Compo G.P. (1998). A practical guide to wavelet analysis. Bull. Am. Meteorol. Soc..

[br0180] Walden A.T., Serroukh A. (2002). Wavelet analysis of matrix-valued time-series. Proc. R. Soc. A, Math. Phys. Eng. Sci..

[br0190] Walter R.K., Reid E.C., Davis K.A., Armenta K.J., Merhoff K., Nidzieko N.J. (2017). Local diurnal wind-driven variability and upwelling in a small coastal embayment. J. Geophys. Res., Oceans.

[br0200] Walter R.K., Stastna M., Woodson C.B., Monismith S.G. (2016). Observations of nonlinear internal waves at a persistent coastal upwelling front. Cont. Shelf Res..

[br0210] Wang Z., Bovik A.C., Sheikh H.R., Simoncelli E.P. (2004). Image quality assessment: from error visibility to structural similarity. IEEE Trans. Image Process..

[br0220] Wu Z., Huang N.E., Long S.R., Peng C.K. (2007). On the trend, detrending, and variability of nonlinear and nonstationary time series. Proc. Natl. Acad. Sci..

[br0230] Yang K., Shahabi C. (2004). A PCA-based similarity measure for multivariate time series. Proceedings of the 2nd ACM International Workshop on Multimedia Databases.

